# Pleiotrophin Commits Human Bone Marrow Mesenchymal Stromal Cells towards Hypertrophy during Chondrogenesis

**DOI:** 10.1371/journal.pone.0088287

**Published:** 2014-02-07

**Authors:** Thibault Bouderlique, Emilie Henault, Angelique Lebouvier, Guilhem Frescaline, Phillipe Bierling, Helene Rouard, José Courty, Patricia Albanese, Nathalie Chevallier

**Affiliations:** 1 CNRS EAC 7149, CRRET Laboratory, Paris-Est University, Créteil, France; 2 EA3952, Cellular and Tissular Bioengineering Laboratory, Paris-Est University, Créteil, France; 3 Cell Therapy Facility, EFS Ile de France, Créteil, France; 4 INSERM UMR955, Paris-Est University, Créteil, France; French Blood Institute, France

## Abstract

Pleiotrophin (PTN) is a growth factor present in the extracellular matrix of the growth plate during bone development and in the callus during bone healing. Bone healing is a complicated process that recapitulates endochondral bone development and involves many cell types. Among those cells, mesenchymal stromal cells (MSC) are able to differentiate toward chondrogenic and osteoblastic lineages. We aimed to determine PTN effects on differentiation properties of human bone marrow stromal cells (hBMSC) under chondrogenic induction using histological analysis and quantitative reverse transcription polymerase chain reaction. PTN dramatically potentiated chondrogenic differentiation as indicated by a strong increase of collagen 2 protein, and cartilage-related gene expression. Moreover, PTN increased transcription of hypertrophic chondrocyte markers such as MMP13, collagen 10 and alkaline phosphatase and enhanced calcification and the content of collagen 10 protein. These effects are dependent on PTN receptors signaling and PI3 K pathway activation. These data suggest a new role of PTN in bone regeneration as an inducer of hypertrophy during chondrogenic differentiation of hBMSC.

## Introduction

Bone formation during growth and regeneration passes through a common process known as endochondral bone formation [1]. In this process, chondrocytes in growth plates proliferate while synthesizing a cartilaginous extracellular matrix (ECM) mainly composed of proteoglycans and collagen 2 (col2). Following differentiation, chondrocytes become hypertrophic. They increase in size by up to ten fold and express specific hypertrophic markers such as matrix metalloprotease 13 (MMP13), collagen 10 (col10) and alkaline phosphatase (ALP). Hypertrophic chondrocytes calcify their surrounding matrix before undergoing apoptosis [2]. Concomitant matrix degradation by MMPs [3] allows vessel ingrowth and invading cells partially degrade the extracellular matrix in preparation for the formation of mature bone by osteoblasts. Osteoblasts deposit an osteoid matrix that will later calcify. Throughout life, bone is submitted to a constant remodeling process, alternating matrix degradation and bone synthesis.

Mesenchymal stromal cells (MSC) from surrounding tissues are the key cellular component of bone regeneration since they can differentiate toward chondrogenic and osteoblastic lineages [4], [5]. During bone and cartilage formation, many growth factors are tightly regulated to give rise to a suitable ECM. The transforming growth factor (TGF) super-family, in particular bone morphogenetic proteins (BMP) and TGF β, are the most known factors involved in this processes [1]. However, numerous other growth factors are also involved in bone and cartilage organogenesis. Among them, Pleiotrophin (PTN) is a 136 amino acid growth factor that has been first isolated from brain and bone matrices [6], [7], [8]. PTN has three known receptors: anaplastic lymphoma kinase (ALK) defined as a high affinity receptor [9], heparan sulfate proteoglycan syndecan 3 (SDC3) and chondroitin sulfate proteoglycan protein tyrosine phosphatase receptor type z (PTPRz), both defined as low affinity receptors [10], [11].

PTN is widely expressed in many tissues during fetal development [12], [13] whereas its post-natal expression is predominantly restricted to nervous system and bone [13]. PTN has been implicated in many processes such as neurite outgrowth during brain development [14] and endothelial cell properties in normal and pathological angiogenesis (for review see [15]). Studies suggest that PTN is involved in the development and regeneration of bone and cartilage because the protein is localized around hypertrophic chondrocytes and osteoprogenitors in rat, mice and chicken developing leg [16], [17], [18]. Moreover, *in vitro* studies showed that PTN treatment stimulates the proteoglycan synthesis of bovine mature chondrocytes [19] and enhances chondrogenesis of chicken limb bud mesenchymal cells in pellet culture [20]. Other *in vitro* experiments showed that PTN induces migration of osteoblast cell lines [18] and osteoprogenitors from human bone marrow [21]. PTN also improves osteoblastic differentiation of bone marrow derived stromal cells [21], [22].

PTN overexpression under the regulation of the osteocalcin promoter in transgenic mice induces a higher bone mineral content and a delayed bone growth rate compared to wild-type mice[23]. However relevance of PTN effects on bone physiology still remains controversial since Lehmann and colleagues showed no specific bone phenotype in PTN deficient mice [24]. Imai and colleagues confirmed that PTN global knockout and wild type mice had the same skeletal morphology although, they observed growth retardation in weight-bearing bones [25]. These data suggest an effect on the growth plate behaviour during growth, thereby indicating an effect of PTN on chondrocytes differentiation. However, no data are available describing a potential effect of PTN on the commitment of chondrocytes to hypertrophy.

The aim of this study was to define the effects of PTN on human bone marrow stromal cell (hBMSC) differentiation towards chondroblastic lineage with a focus on chondrocyte hypertrophy. We evaluated chondrocytic commitment of hBMSC in presence of recombinant human PTN by histological and quantitative reverse transcription polymerase chain reaction analysis. We then focused on hypertrophic differentiation and established a potential new role of PTN during endochondral bone formation.

## Materials and Methods

### Human Bone Marrow Stromal Cell Isolation

**Figure 1:**
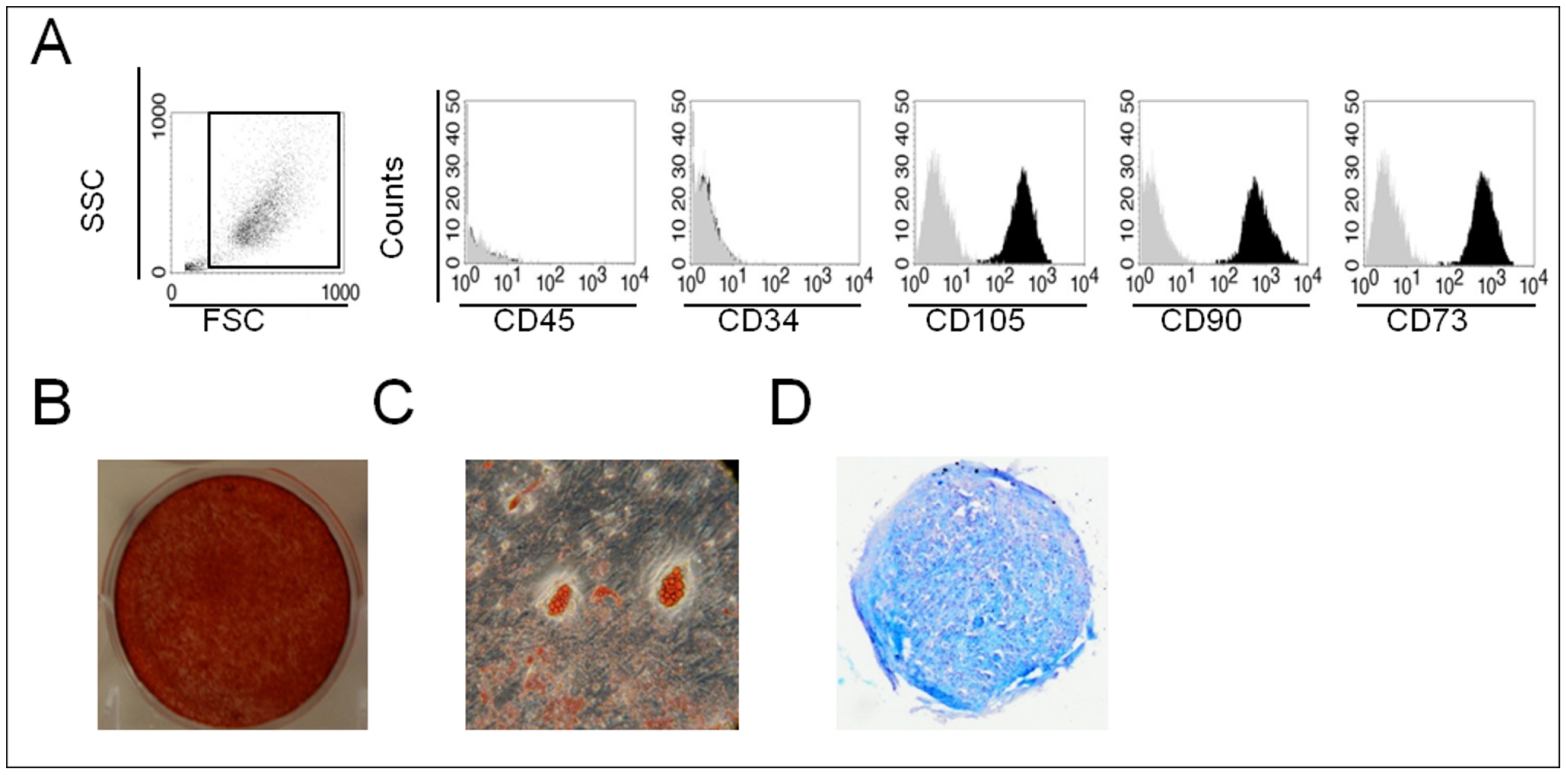
Characterization of hBMSC. hBMSC from 3 independent patients were characterized after their isolation from bone marrow. (**A**): FACS analysis for CD45-FITC, CD34-FITC, CD105-PE, CD90-FITC and CD73-PE markers (black histogram) and their corresponding isotype (grey histogram), (**B**): Alizarin red staining, (**C**): Oil red O staining, (**D**): Alcian Blue staining.

Human bone marrow was obtained from iliac crest marrow aspirates of patients undergoing standard bone marrow transplantation procedures (Henri Mondor Hospital, AP-HP Créteil, France) after signature of an informed consent form. Researchers did not take part in collecting these samples. Anonymous sample (3- to 5-ml volumes) from three healthy donors (26–38 years old) were provided to EA3952 for the purpose of research only, under the European project REBORNE, grant agreement number N°IDRCB: 2011-A00797-34 and ethical committee approval N°CPP: 2011-R28. Nucleated cells from fresh marrow were seeded at a density of 2.10^5^ cells/cm^2^ in 225-cm^2^ flasks. A cell sample was used for the confirmation of hBMSC characteristics as previously described [26]. Briefly, all the hBMSC were positive for CD90, CD105, CD73, and negative for CD34 and CD45, and were able to differentiate into osteogenic, chondrogenic and adipogenic lineages (Figure 1). hBMSCs were expanded in α-modified Eagle’s medium (αMEM) (Life technologies, Cergy Pontoise, France) containing 10% lot-selected fetal bovine serum (FBS) (StemCell Technologies, Grenoble, France) supplemented with 0,5% Cifloxacin (Bayer Pharma, Puteaux, France). Culture medium was changed twice every week and cultures were maintained in a humidified atmosphere with 5% CO_2_ at 37°C. When cells reached 80–90% confluence (passage zero, P0), they were detached using trypsin/EDTA (PAA Laboratories, Austria) and then reseeded at 10^3^ cells/cm^2^ (passage one, P1).

### Characterisation and Differentiation of Human BMSC

The capacity of hBMSC to differentiate into the osteogenic and adipogenic lineages was determined. For this purpose, cells were seeded in 6-well plates. For osteogenic differentiation, at 25% confluence the media was supplemented with 50 µM ascorbic acid-2-phosphate, 10 mM β-glycerophosphate and 0,1 µM dexamethasone (Sigma, Saint Quentin Fallavier, France). On day 21, the monolayers were fixed in 70% ethanol for 1 h at 4°C and stained for 15 min with alizarin red-S (Sigma) at room temperature (RT). For adipogenic differentiation, at 80% confluence the media was replaced by a high glucose medium (Invitrogen) supplemented with 10% FBS, 0,1 µM dexamethasone, 0,2 mM indomethacin, 0,01 mg/ml insulin and 0,5 mM IBMX. On day 21, the monolayers were fixed using 4% paraformaldehyde for 5 min at RT, and then stained for 15 min with 0,3% oil-red O (Sigma)/60% isopropanol.

### Flow Cytometry

hBMSCs (P1) from three BM were resuspended in phosphate buffer containing 2% FBS with fluorescein isothiocyanate (FITC)- or phycoerythrin (PE)-coupled antibodies against CD105 (Caltag Laboratory, CA, USA), CD90, CD73, CD34 or CD45, or the corresponding mouse IgG1 isotype (all from Becton Dickinson and Company, Franklin Lakes, NJ, USA) for 15 min at RT. The cells were washed and examined using a FACScan flow cytometer. The data were analysed using the Cell Quest software (Becton, Dickinson and Company). Positive expression was defined as fluorescence greater than 95% of that of the corresponding isotype-matched control antibodies.

### Chondrogenic Differentiation

Differentiations were performed at passage two. Chondrogenic differentiation was performed in pellet culture using the Stempro Chondrogenesis Differentiation Kit (Life technologies), as described by manufacturer, with increasing doses of PTN. Briefly, 3×10^5^ hBMSC were seeded in V-bottomed wells and centrifugated to form a pellet. Medium was removed and cells incubated for three hours in a humidified atmosphere with 5% CO_2_ at 37°C. The pellet was detached by adding differentiation medium. Medium was changed twice a week until analysis. On days 0, 7, 14, pellets were lysed for RNA extraction. On day 14, pellets were frozen to perform sulfated glycosaminoglycans (GAG) extraction as previously described [27]. GAG quantification was conducted using five pellets per condition for three patients, and normalized by pellet volume. On day 21, pellets were fixed in 4% formaldehyde for further histological analysis. The same chondrogenic differentiation protocol was applied in the presence of 15 µM Ly294002 (a potent inhibitor of PI3K; Sigma) or 100 ng/ml P111-136 peptide (corresponding to the C-terminal domain of PTN; LTKPKPQAESKKKKKEGKKQEKMLD; Altergen, Schiltigheim, France) from day 0 to day 14. Chondrogenic differentiation was assessed at days 14 and 21 by qRT-PCR analysis and histological analysis respectively.

### Quantitative Real-time Reverse Transcription–polymerase Chain Reaction (qRT-PCR)

Total mRNA from chondroblastic cultures was isolated using TRIzol® reagent (Life technologies) respectively, as described by manufacturers. DNase (Promega, France)-treated RNA were reverse transcribed with RT Superscript III (Life technologies), cDNA real-time amplification was performed with FastStart Universal SYBR Green Master (Rox; Roche, Meylan, France) following the manufacturer’s instructions and monitored with the ABI Prism 7500 Sequence Detection System (PerkinElmer/Applied Biosystem, Rotkreuz, Switzerland). Primers (Table 1) were designed by Primer3output software [28] and obtained from Eurofins MWG (Huntsville, Germany). Primer efficiency between 95% and 100% was ensured. Results were normalized to GAPDH (ΔCT = CT_gene of interest_ -CT_GAPDH_) and are reported as relative gene expression (2^-ΔCT^).

**Table 1:** Primer sequences.

Gene	Primer sequence	Accession number	Product size
**Housekeeping gene**			
Glyceraldehyde-3-phosphate	forward 5'-TGC CTG ATG AGA CAG AGG TG-3'	NM_0020463	97
dehydrogenase (GAPDH)	reverse 5'-TCC ACC TGG ACA GGA TTA GC-3'		
**Chondroblastic markers**			
SRY-box9 (Sox9)	forward 5'-AGA CAG CCC CCT ATC GAC TT-3'	NM_000346.3	108
	reverse 5'-CGG CAG GTA CTG GTC AAA CT-3'		
Collagen 9A1 (col9)	forward 5'-GCA GGT TTG CAT GAG AGT CA-3'	NM_078485.3	142
	reverse 5'-TGG GAA ACC ATT CTC TCC AG-3'		
Cartilage oligomeric matrix protein (COMP)	forward 5'-ACA ATG ACG GAG TCC CTG AC-3'	NM_000095.2	115
	reverse 5'-TCT GCA TCA AAG TCG TCC TG-3'		
**Proteoglycan core**			
Aggrecan (ACAN)	forward 5'-AGG AGT CCC TGA CCT GGT TT-3'	NM_001135.3	108
	reverse 5'-TTC AAC CAA ACT GGT GTC CA-3'		
Biglycan (BGN)	forward 5'-GGA CTC TGT CAC ACC CAC CT-3'	NM_001711.4	159
	reverse 5'-AGC TCG GAG ATG TCG TTG TT-3'		
Decorin (DCN)	forward 5'-GGA CCG TTT CAA CAG AGA GG-3'	NM_133507.2	147
	reverse 5'-GAC CAC TCG AAG ATG GCA TT-3'		
Versican (VCAN)	forward 5'-GGT GCA CTT TGT GAG CAA GA-3'	NM_001119808.1	159
	reverse 5'-TTC GTG AGA CAG GAT GCT TG-3'		
**Hypertrophic markers**			
Alkaline phosphatase (ALP)	forward 5'-CCA CGT CTT CAC ATT TGG TG-3'	NM_000478.4	96
	reverse 5'-GCA GTG AAG GGC TTC TTG TC-3'		
Matrix metalloproteinase 13 (MMP13)	forward 5'-TTG AGC TGG ACT CAT TGT CG-3'	NM_002427.3	172
	reverse 5'-GGA GCC TCT CAG TCA TGG AG-3'		
Collagen 10A1 (col10)	forward 5'-GCT AAG GGT GAA AGG GGT TC-3'	NM_000493.3	118
	reverse 5'-CTC CAG GAT CAC CTT TTG GA-3'		
**PTN receptors**			
Anaplastic lymphoma	forward 5'-GCC AGA AAC TGC CTC TTG AC-3'	NM_004304.4	90
receptor tyrosine kinase (ALK)	reverse 5'-GCT CGC CCT GTA GAT GTC TC-3'		
Protein tyrosine phosphatase	forward 5'-CCC CAA CAA GAG GAA GTG AA-3'	NM_002851.2	80
receptor type z (PTPRz)	reverse 5'-AGT GAC TGG TTG GGA AGT GG-3'		
Syndecan 3 (SDC3)	forward 5'-CCA GAG ACC TTC CTG ACC AC-3'	NM_014654.3	85
	reverse 5'-CTT CTG GCA GCT CGA AGT CT-3'		

### Histological Analysis

Chondrogenic pellets were fixed in 4% formaldehyde (Sigma Aldrich) and embedded in paraffin. Sections (3 µm) were stained with Alcian blue 8GX and counterstained with hematoxylin (both from Sigma Aldrich), or stained with alizarin red-S alone. Immunohistochemical staining for collagen 2 (clone 6b3; MerckMillipore Saint-Quentin en Yveline, France) and collagen 10 (Sigma Aldrich) was carried out after antigen retrieval with boiling citrate buffer for two minutes (Dako, Trappes, France) and incubation with primary antibody (1/100) overnight at 4°C. Antibody detection was performed using a goat anti-mouse multiHRP (MerckMillipore) and histogreen (Eurobio-Abcys, Les Ulis, France), sections were counterstained with hematoxylin (Sigma Aldrich).

### Statistical Analysis

Results were expressed as mean of values ± standard error mean (SEM) from three independent patients with at least three to five values per condition and per patient. Statistical analyses were performed using a one-way ANOVA Kruskall-Wallis test (GraphPad Software). Differences between groups with a p-value of ≤0.05 were considered to be significant.

## Results

### Characterization of hBMSC

Before further analysis, hBMSC were analysed for mesenchymal stromal cells surface markers and differentiation properties (Figure 1). hBMSC are negative for CD45 and CD34 and positive for CD105, CD90 and CD73 (Figure 1A). hBMSC are able to differentiate towards osteoblastic phenotype (Figure 1B), adipocyte lineage (Figure 1C) and chondrocytic lineage (Figure 1D).

### PTN Potentiates Chondrogenic Differentiation of hBMSC

**Figure 2:**
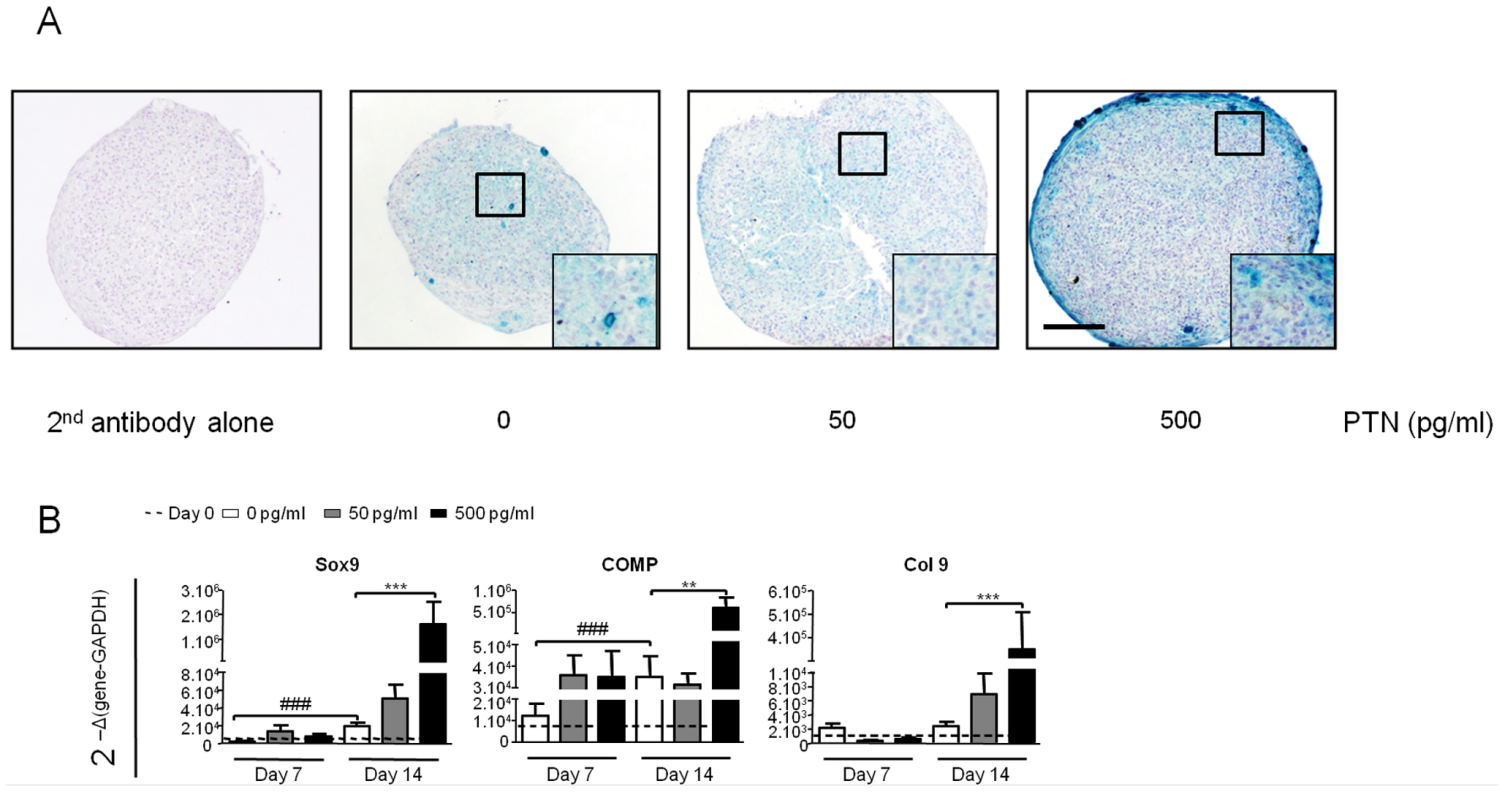
PTN increases cartilage specific protein and gene expression during hBMSC chondrogenic differentiation. hBMSC from 3 independent patients were cultured in micromass with chondrogenic medium in absence or with increasing doses of PTN (0 pg/ml white boxes, 50 pg/ml grey boxes and 500 pg/ml black boxes) for 21 days. All conditions were performed in triplicate per patient. (**A**): Collagen 2 immunostaining of chondrogenic pellets. Side box shows an enlargement (x3) of the black square. Bars represent 100 µm. (**B**): Real-time polymerase chain reaction analysis of cartilage related genes expression. RNA were purified from hBMSC at day 0 and after 7 and 14 days of culture without or with PTN. Expression levels of cartilage genes: SRY-box9 (Sox9), Cartilage Oligomeric Matrix Protein (COMP) and Collagen9A1 (Col9) are normalized to Glyceraldehyde 3-Phosphate Dehydrogenase (GAPDH). Values are the mean±SEM. The gene expression values at day 0 are represented by straightened lines. Statistical analysis were performed, with a one way anova Kruskal-Wallis test, between values from free-PTN conditions (white boxes) at day 0, 7 and 14 (##: p<0.01; ###: p<0.001), and between values from increasing PTN doses from the same day (**: p<0.01; ***: p<0.001).

Chondrogenic potential of PTN was tested using hBMSC cultured in pellets with chondroinductive medium (CM). Pellet slices were immunostained for col2 at day 21 (Figure 2A). No staining was observed in the presence of the secondary antibody alone. In the absence of PTN, CM pellets showed a minimal staining and same results were obtained on pellets treated with 50 pg/ml of PTN. Surprisingly pellets treated with 500 pg/ml of PTN had strong col2 staining compared to other conditions. Using microscopy at high magnification indicates chondrocytes within a col2-rich extracellular matrix (Figure 2A). Chondrogenic marker expression was further analyzed by qRT-PCR at days 0, 7 and 14 (Figure 2C). Between day 0 and day 7 the transcription of Sox9, COMP and col9 genes was not modified in CM alone. Between day 7 and day 14, CM condition was associated with an up regulation of Sox9 (8-fold) and COMP (3-fold) expressions. PTN at 50 pg/ml had no significant effect on chondrogenic gene expression as compared to CM alone. In contrast, addition of 500 pg/ml of PTN induced a large increase in the expression of Sox9 (600-fold), collagen 9 (160-fold) and COMP (50-fold) at day 14, as compared to CM alone.

### PTN Stimulates Proteoglycan Synthesis During Chondrogenic Differentiation of hBMSC

**Figure 3:**
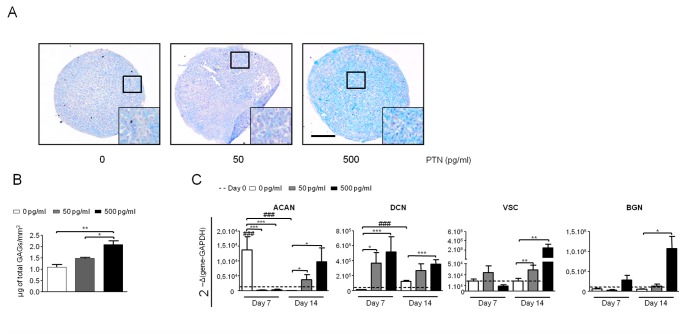
PTN increases glycosaminoglycans content of chondrogenic induced hBMSC. hBMSC from 3 independent patients were cultured in micromass with chondrogenic medium in the absence or with increasing doses of PTN (0 pg/ml white boxes, 50 pg/ml grey boxes and 500 pg/ml black boxes) for 21 days. All conditions were performed in triplicate per patient. (**A**): Alcian blue staining of sulfated GAGS in chondrogenic pellets. Side box shows an enlargement (x3) of the black square. Bars represent 100 µm. (**B**): Total sulfated GAGs quantification. After 14 days in micromass culture, sulfated GAGs were extracted from pellets and quantified as previously described [27]. GAG amount was normalized according to pellet volume and reported as µg of total GAGs per mm^3^. (**C**): Real-time polymerase chain reaction analysis of proteoglycan protein core-gene expression. RNA were purified from hBMSC at day 0 and after 7 and 14 days of culture without or with PTN. Expression levels of proteoglycans core expression Aggrecan (ACAN), Biglycan (BGN), Decorin (DCN), Versican (VCAN) are related to Glyceraldehyde 3-Phosphate Dehydrogenase (GAPDH). Values are the mean±SEM. The gene expression values at day 0 are represented by straightened lines. Statistical analysis were performed, with a one way anova Kruskal-Wallis test, between values from free-PTN conditions (white boxes) at day 0, 7 and 14 (###: p<0.001), and between values from increasing PTN doses from the same day (*: p<0.05; **: p<0.01; ***: p<0.001).

GAG synthesis is of major importance in cartilage by giving it compressive strength and participating to organize collagen fibrils [29]. We evaluated the effects of PTN on sulfated GAG amount in hBMSC pellets by alcian blue staining at day 21. In the absence of PTN, pellets showed little alcian blue staining. However, when pellets were treated with PTN they were strongly stained, (especially with the 500 pg/ml dose) indicating high GAG content. When the pellets were viewed at high magnification, cells could be seen in lacunae surrounded by GAG–rich extracellular matrix, proving effective chondrogenic differentiation (Figure 3A). Quantification of GAG content confirmed our histological observations (Figure 3B) since PTN treated pellets (500 pg/ml) had a significant two-fold increase in sGAG amount compared to CM alone. GAG chains bind to protein cores within the ECM. The expression of core proteins aggrecan (ACAN), decorin (DCN), versican (VCAN) and biglycan (BGN) were assessed by qRT-PCR at days 0, 7 and 14. In the absence of PTN, CM significantly enhanced ACAN expression at day 7 and DCN expression at day 14 whereas no effects were observed on VCAN and BGN expression (Figure 3C). ACAN expression was drastically decreased at day 14 in CM. ACAN expression at day 7 was decreased, with both 50 pg/ml and 500 pg/ml PTN treatments, whereas a clear induction of expression was observed at day 14 as compared to CM alone. This result suggests that PTN could be involved in modulation of ACAN expression during chondrogenic differentiation. PTN treatments induced an increase of DCN expression from day 7 to day 14. Compared to CM alone, PTN treatments significantly augmented expression of VCAN at day 14. Finally, 500 pg/ml PTN treatment strongly induced expression of BGN at day 14. These data clearly indicate an inductive effect of PTN on proteoglycan core expression during chondrogenic differentiation.

### PTN Induces Hypertrophy in Chondro-induced hBMSC

**Figure 4:**
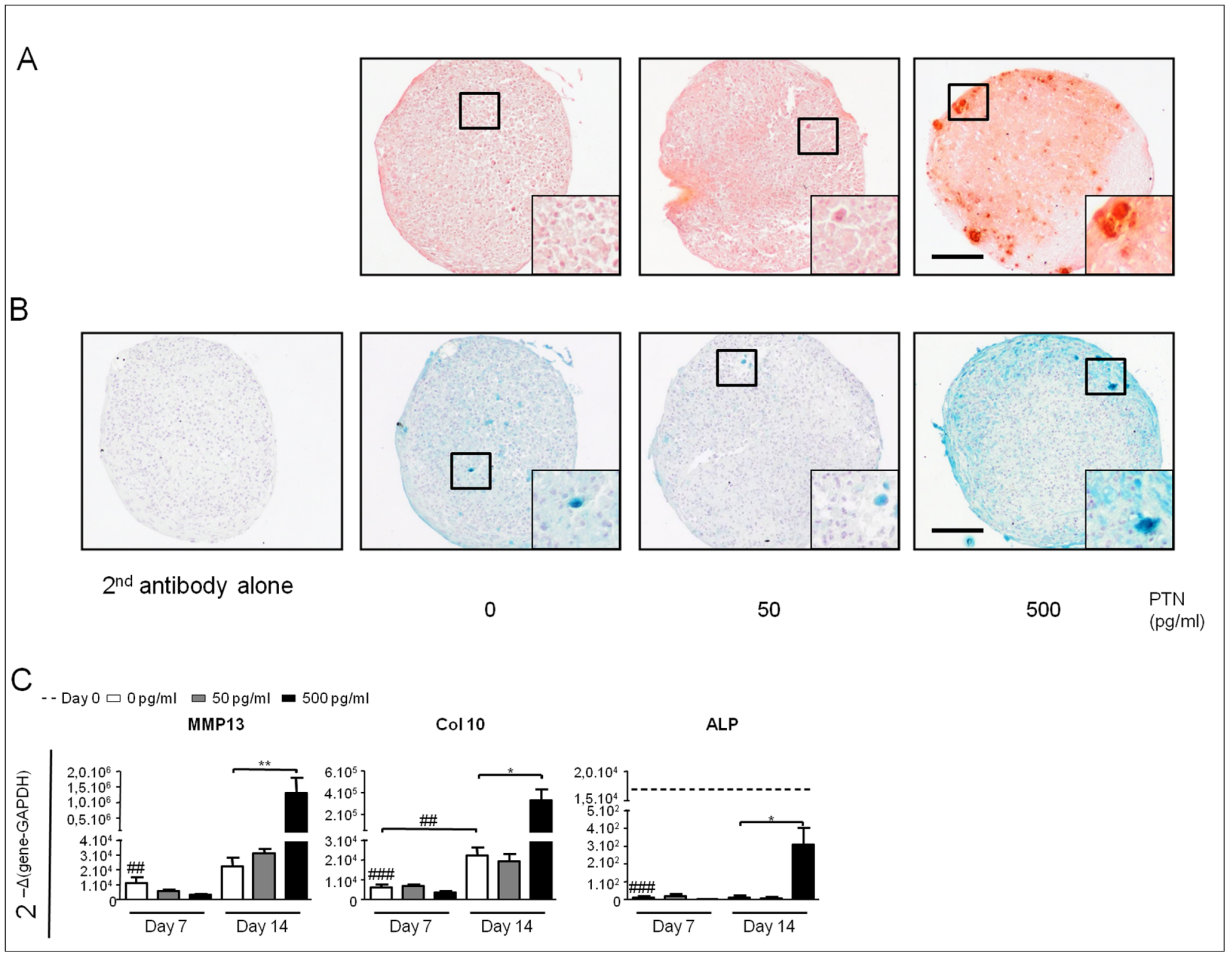
PTN induces hypertrophic differentiation of hBMSC. hBMSC from 3 independent patients were cultured in micromass with chondrogenic medium in absence or with increasing doses of PTN (0 pg/ml white boxes, 50 pg/ml grey boxes and 500 pg/ml black boxes) for 21 days. All conditions were performed in triplicate per patient. (**A**): Alizarin red staining of chondrogenic pellets. (**B**): Collagen10 immunostaining of chondrogenic pellets. Side box shows an enlargement (x3) of the black square. Bars represent 100 µm. (**C**): Real-time polymerase chain reaction analysis of hypertrophic related genes expression. RNA were purified from hBMSC at day 0 and cultured without or with PTN at day 7 and 14. Expression levels of hypertrophic genes Matrix Metalloprotease 13 (MMP13), Collagen 10A1 (Col10) and Alkaline Phosphatase (ALP) are related to Glyceraldehyde 3-Phosphate Dehydrogenase (GAPDH). Values are the mean±SEM. The gene expression values at day 0 are represented by straightened lines when they are different of 0. Statistical analysis were performed, with a one way anova Kruskal-Wallis test, between values from free-PTN conditions (white boxes) at day 0, 7 and 14 (##: p<0.01; ###: p<0.001) and between values from increasing PTN doses from the same day (*: p<0.05; **: p<0.01).

Next, we investigated the effects of PTN on the hypertrophic differentiation of chondro-induced hBMSC. We stained pellets with alizarin red-S (Figure 4A) and for col10 (Figure 4B) at day 21. Pellets treated with 500 pg/ml of PTN showed an intense red staining with hot-spots around hypertrophic cells, whereas the lower PTN dose (50 pg/ml) and CM medium alone, had minimal staining (Figure 4A). Mineralizing cells were mainly found at the edge of the pellet. Higher power magnification of the alizarin red-S -positive area indicated that these cells may be undergoing physiological hypertrophy because the cells were larger in comparison with those around them. Furthermore, pellets treated with 500 pg/ml of PTN showed an intense staining for col10 whereas the two others conditions showed only basal levels. Staining was not observed in the sole presence of the secondary antibody (Figure 4B). We then analyzed expression of hypertrophic markers by qRT-PCR on days 0, 7 and 14. MMP13 and col10 were not detected at day 0. CM induced MMP13 and col10 expression by day 7 and day 14, while ALP expression decreased during chondrogenic induction. We observed that whereas 50 pg/ml of PTN had no effect as compared to CM alone, 500 pg/ml of PTN strongly increased expression of MMP13 (60-fold) and col10 (15-fold) at day 14 as compared to CM alone. Moreover, ALP expression was induced at day 14 by PTN treatment at 500 pg/ml (Figure 4C). These data clearly show an inductive effect of PTN on the commitment of hBMSC toward a hypertrophic state.

### The Expression of PTN Receptors are Modulated during Chondrogenic Differentiation

**Figure 5:**
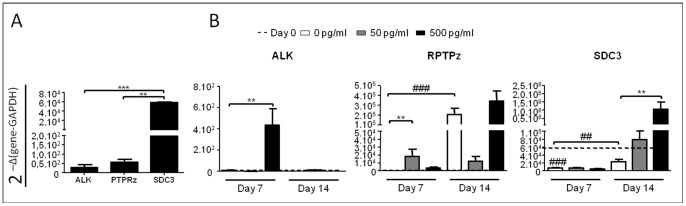
PTN receptors are expressed on undifferentiated hBMSC and PTN modulates their expression during chondrogenic differentiation. hBMSC from 3 independent patients were cultured in micromass with chondrogenic medium in absence or with increasing doses of PTN (0 pg/ml white boxes, 50 pg/ml grey boxes and 500 pg/ml black boxes). All conditions were performed in triplicate per patient. (A): Real-time polymerase chain reaction analysis of PTN receptors genes expression before differentiation induction. Expression levels of Anaplastic Lymphoma receptor tyrosine Kinase (ALK), Protein Tyrosine Phosphatase Receptor type z (PTPRz) and Syndecan3 (SDC3) are related to Glyceraldehyde 3-Phosphate DeHydrogenase (GAPDH). (B): Real-time polymerase chain reaction analysis of PTN receptors genes on hBMSC during chondrogenic differentiation at days 7 and 14. Values are the mean±SEM. The gene expression values at day 0 are represented by straightened lines. Statistical analysis were performed, with a one way anova Kruskal-Wallis test, between values from free-PTN conditions (white boxes) at day 0, 7 and 14 (##: p<0.01; ###: p<0.001), and between values from increasing PTN doses from the same day (**: p<0.01).

As we had shown that PTN plays a role in chondrogenic hypertrophic differentiation of hBMSC, we investigated the expression profiles of PTN receptors by qRT-PCR analysis during hBMSC chondrogenic differentiation. The three receptors are expressed in hBMSC before differentiation, with a higher gene expression of SDC3 compared to PTPRz and ALK (Figure 5A). We then analyzed their expression patterns during chondroinduction of hBMSC in CM conditions. Whereas ALK expression was not modulated during chondrocytic differentiation in the absence of PTN, its expression was strongly and transiently induced at day 7 by the 500 pg/ml dose of PTN. In contrast, PTPRz expression was induced by CM treatment at day 14 and no significant modulation was observed with 500 pg/ml of PTN. Surprisingly PTPRz expression was prematurely induced at day 7 with 50 pg/ml of PTN but did not increase at day 14. Finally, SDC3 expression was down regulated during chondroinduction at day 7 and 14 but was strongly up regulated by PTN treatments with a significant increase for 500 pg/ml at day 14. Our data indicate the presence of the three known PTN receptors on hBMSC and the modulation of their expression during hBMSC chondrogenesis.

### The Chondroinductive Effects PTN are Dependent on PTN Receptor and Pi3K Signaling

Receptors ALK and PTPRz have previously been shown to activate the PI3K pathway [9], [30], which is involved in chondrocyte hypertrophy [31]. However, there is currently no link between ALK or PTPRz and chondrocyte hypertrophy. We investigated the implication of these receptors by using a peptide corresponding to PTN C-terminal domain (P111-136), as a competitive inhibitor for PTN signaling through ALK and PTPRz. Involvement of the PI3K pathway in the PTN-induced hypertrophy was also tested using Ly294002, a potent inhibitor of PI3K.

**Figure 6:**
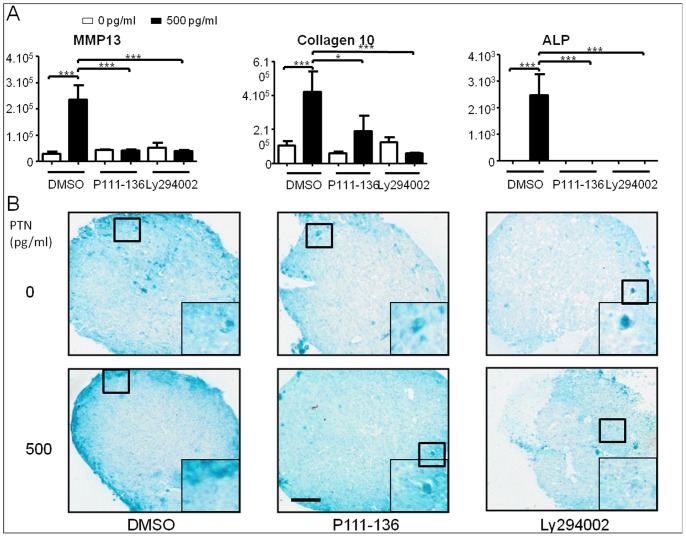
PTN chondroinductive effects are inhibited by inhibitors of PTN receptors and Pi3K. hBMSC were cultured in micromass with chondrogenic medium with 0/ml or 500 pg/ml of PTN with or without Ly294002 (15 µM) or p111-136 peptide (100 ng/ml). All conditions were performed in triplicate. (**A**): Real-time polymerase chain reaction analysis of late chondrogenic marker genes after 14 days of treatment. Expression levels of Matrix Metalloprotease 13 (MMP13), Collagen 10A1 (Col10) and Alkaline Phosphatase (ALP) are related to Glyceraldehyde 3-Phosphate Dehydrogenase (GAPDH). Values are the mean±SEM. Statistical analysis were performed, with a one way anova Kruskal-Wallis test between DMSO treated and Ly294002 or P111-136 treated hBMSC (*: p<0.05; ***: p<0.001). (**B**): Collagen10 immunostaining of chondrogenic pellets. Side box shows an enlargement (x3) of the black square. Bars represent 100µm.

We cultured hBMSC under chondrogenic conditions, with PTN at 500 pg/ml or vehicle (DMSO) in the presence or absence of the inhibitors. Treatment with DMSO did not modify the effect of PTN on the expression of hypertrophy-related genes (Figure 6A and 4C). In the absence of PTN, P111-136 and Ly294002 did not modify the expression of these genes. However, in the presence of PTN, both P111-136 and Ly294002 treatments resulted in a return to the baseline expression of MMP13, col10 and ALP. As previously described, PTN induced a higher accumulation of col10 in the ECM, mainly at the edge of the pellet (Figure 6B and 4B). However, Ly294002 and P111-136 treatments resulted in a decreased col10 immunostaining in presence of exogenous PTN, and surprisingly also in its absence (Figure 6B). These data indicate that chondrogenic induction by PTN passes at least in part through ALK and PTPRz and further PI3K activation.

## Discussion

Bone formation and repair can be obtained by a direct intramembraneous bone formation or by an endochondral formation, involving a cartilage template prior to ossification. These processes involve complex extracellular matrix protein deposition and growth factor expression. Among them PTN is a heparin-binding growth factor expressed in the growth plate of mice during pre-natal and post-natal bone formation. Its expression is down regulated during adulthood but is induced in the callus during bone healing [17], [32]. Moreover, human patients with fracture union showed higher seric levels of PTN than patients with delayed union [33]. We hypothesized here that PTN effect in bone could be due in part to its activity on chondrogenic differentiation during endochondral bone formation. We then decided to test the effect of PTN during chondroblastic differentiation of hBMSC.

We show that higher doses of PTN (500 pg/ml) enhances the expression of cartilage specific protein (Sox9, col2, col9 and COMP), proteoglycan core (BGN, DCN and VCAN) and GAG synthesis during chondrogenic differentiation of hBMSC. Previous data had already indicated that PTN potentiates late chondrogenic marker expression such as col2 and modulates GAG synthesis and expression of proteoglycan core proteins such as BGC and ACAN [19]. However, these data were obtained using mature chondrocytes from articular cartilage whereas our results indicate for the first time that PTN can have a chondrogenic differentiation potential even on immature adult hBMSC.

Previous studies indicated that the PTN protein is present in hypertrophic zone of growth plate and callus in mice, rat and chicken [16], [17], [32], expressed by chondroblasts and osteoblasts. Our results demonstrate that PTN enhances hypertrophic markers expression such as MMP13, col10 and ALP during chondrogenic differentiation of hBMSC. This suggests that PTN is involved in chondrocytic hypertrophy, a key step of endochondral bone formation [2]. It remains to be elucidated if PTN acts alone to induce hypertrophy or if it mediates hypertrophy through interactions with other growth factors such as TGF β or BMP that were previously described as inducers of chondrogenesis and to act synergistically with PTN [1], [34].

Previous results obtained by Tapp and colleagues showed that PTN inhibits mature chondrocytes proliferation *in vitro* [19]. As we showed that PTN induces chondrogenic hypertrophy of hBMSC, we hypothesize that the growth retardation observed in PTN over-expressing mice [23] could be due to chondrocytes leaving the proliferating zone and becoming prematurely hypertrophic. Surprisingly, the absence of PTN also results in growth retardation in young PTN-knockout mice [25]. As we showed that PTN induces chondrocytic hypertrophy, this could be explained by a reduced hypertrophy of chondrocytes in PTN knock out mice.

The analysis of the gene expression profiles of PTN receptors indicates that the three known receptors ALK, PTPRz and SDC3 are expressed by hBMSC. SDC3 has the higher expression compared to PTPRz and ALK that are very low in these undifferentiated cells. As Seghatoleslami et al. have previously showed that blocking SDC3 with an antibody inhibits chondrogenic differentiation [35], we can hypothesize that the chondrogenic differentiation of hBMSC observed after PTN addition is mediated in part through SDC3 signalling. PTN is expressed in the callus [32]. As SDC3 has also been shown to be involved in osteoblasts migration [18], the expression of SDC3 by hBMSC could allow them to be recruited on site via the PTN/SDC3 pathway where they can play a role in callus formation [4].

Chondrogenic differentiation of hBMSC was associated with an up-regulation of PTPRz and a down-regulation of SDC3, whereas ALK expression was not induced. This shows that these two proteoglycans are modulated during chondrogenesis and may play a role in this commitment. PTPRz has already been involved in bone growth through its expression by osteoblasts [36], however no data are available on its link with chondrogenesis. hBMSC chondrogenic differentiation is associated with an up-regulation of PTPRz expression. PTPRz is a constitutive phosphatase involved in activation of the β-catenin pathway [37]. As β -catenin is essential for cartilage and bone development [38], it would be of interest to test if PTPRz regulation could be involved in chondrogenic differentiation through the β-catenin pathway.

The specific induction of ALK and SDC3 by PTN in the chondrogenic medium may suggest their involvement on the hBMSC hypertrophic commitment. Increased expression of ALK associated to its activation by PTN ligand could allow PI3K/Akt pathway activation [9], which is involved in chondrocyte hypertrophy and ECM mineralization [31], [39]. PTPRz expression is not modulated by PTN, nevertheless its interaction with PTN inhibits its phosphatase activity [40] which may increase phosphorylation level of ALK and PI3K/Akt pathway activation.

We can hypothesize that during chondrogenic differentiation, PTPRz and ALK activities are in balance for controlling chondrogenic and hypertrophic phenotypes. The inhibition of PTN effect on hypertrophic differentiation by the P111-136 peptide and Ly294002 indicates that PTN effects pass at least through ALK or PTPRz, and PI3K activation. In the absence of exogenous PTN, these two inhibitors also had an inhibitory effect on a basal col10 protein accumulation. This suggests that during chondrogenic differentiation hBMSC could start to synthesize PTN that could have induced col10 synthesis. These data suggest that adding exogenous PTN at the beginning of the culture enhanced the chondroinduction and accelerates the differentiation toward hypertrophic state, whereas in absence of exogenous PTN, hypertrophic differentiation will appear later mainly because of endogenous PTN synthesis.

Finally, it has been shown that SDC3 is expressed by hypertrophic chondrocytes in growth plate and during osteoarthritis [41], however, its role is not known in these processes. As SDC3 was involved in cytoskeleton organization through the Src/cortactin pathway [42], it will be of interest to study this pathway in chondrocyte shape control during hypertrophy induction.

## Conclusion

Our findings show that PTN treatment potentiates chondrogenic differentiation of hBMSC and induces their hypertrophy highlighting a new role of PTN in bone metabolism. This new PTN effect could be an important cue in the shift of chondrocytes toward hypertrophy observed in the callus during bone regeneration. The angiogenic role of PTN may be an additional advantage in producing a physiological repair in bone lesion using a scaffold containing PTN and hBMSC.
